# Successful Laparoscopic-Assisted Supracervical Hysterectomy in a 14-Week-Sized Fibroid Uterus: An Autobiographical Case Report

**DOI:** 10.7759/cureus.21543

**Published:** 2022-01-24

**Authors:** Leah Eburne, Richard J Vagovic

**Affiliations:** 1 Psychiatry, Florida State University College of Medicine, Daytona Beach, USA; 2 Obstetrics and Gynecology, Florida State University College of Medicine, Daytona Beach, USA

**Keywords:** lgbtq, african american/black, case report, autobiographical case report, morcellation, lash, hysterectomy, endometriosis, fibroid uterus, leiomyoma

## Abstract

Uterine leiomyomas are a very common gynecological condition. This case report describes the author’s experience pursuing definitive treatment of a 14-week-sized fibroid uterus, the associated menometrorrhagia, and bulk symptoms. Barriers still remain if women of reproductive age prefer ovary-sparing hysterectomy. Laparoscopic supracervical hysterectomy with contained morcellation remains a well-tolerated and low-risk option dependent on surgeon expertise and informed patient consent. Literature review reveals the psychosocial and financial burden of uterine leiomyomas may present multiple challenges, affecting the quality of life.

## Introduction

Leiomyomas, commonly known as uterine fibroids or myomas, are a prevalent and benign gynecological condition affecting >70% of white women and >80% of African American women [1]. Besides African ancestry, other risk factors include early menarche, nulliparity, family history, age up to menopause, and obesity [1,2]. Presentation is highly variable and may involve no symptoms at all. Symptoms prompting patients to seek evaluation include heavy and prolonged uterine bleeding, infertility, pain with penetrative intercourse, and issues commonly associated with the distorting relationship between the fibroid uterus and other pelvic organs such as urinary dysfunction, constipation, back pain, and noncyclic pelvic pain [1,2]. Diagnosis may be made on routine pelvic screening with a fibroid uterus being palpated upon exam. Ultrasound confirmation remains the primary diagnostic tool as it is low cost, low risk, and widely accessible [1-3]. Saline-infused sonohysterography and magnetic resonance imaging (MRI) may serve as adjuncts particularly for detailed visualization of the pelvic landscape to rule out other pathology and for surgical planning [3].

There are many pharmacologic and non-pharmacologic management options available for symptomatic patients. Pharmacologic treatment involves non-steroidal anti-inflammatory drugs (NSAIDs), tranexamic acid, and hormonal therapy such as progesterone suppression, combination oral contraceptives, intrauterine devices, selective estrogen receptor modulators, and gonadotropin-releasing hormone (GnRH) analogs [1-3]. Non-pharmacologic treatment is largely procedure-based, which is divided into minimally invasive and surgical interventions. This includes uterine artery embolization, high-intensity focused ultrasound, myomectomy, and hysterectomy. Each procedure carries its own risks and may be limited by the ability to preserve fertility, myoma size and number, and patient habitus [4,5]. Mass recurrence and further surgical treatment are a consideration for all procedures but hysterectomy [1-4]. Hysterectomy remains the definitive and most common intervention but is highly dependent on surgeon expertise and patient preference [1-3]. Due to earlier presentation, increased rate of myoma growth, and overall severity of disease, African American women possess higher rates of myomectomy and hysterectomy in the United States [4].

## Case presentation

I was diagnosed in 2017 while modeling for a focused assessment with sonography in trauma (FAST) ultrasound course. I was a full-time overnight shift emergency department scribe living paycheck to paycheck with no primary care physician or gynecologist. I could afford only catastrophic health insurance. Until that point in time, I did not presume there was anything amiss. I was 33 years old, G0P0, queer, and not sexually active. The changes in my body laid insidiously buried while I was busy working six days a week and applying to medical school. My 28-day cycles were always regular, I did have pre-menstrual irritability, and it was not unusual to go through eight to nine pads on the first day of my menses. Using half a 48-count pack of super-absorbent overnight pads in a seven-day period was my normal. Some cycles were worse than others and this was my normal. I would arise from sleep before bleeding through the sheets and go about life with a cup(s) of coffee and a full schedule.

Soon after, I established with a gynecologist. The exam was positive only for obviously palpable uterine leiomyomas, and I was placed on oral Aygestin R (Norethindrone 5 mg) to be taken daily. It was hoped that this high dose could shrink or mitigate further fibroid growth while being referred out to another gynecologist, a skilled laparoscopic surgeon and reproductive endocrinologist.

**Figure 1:**
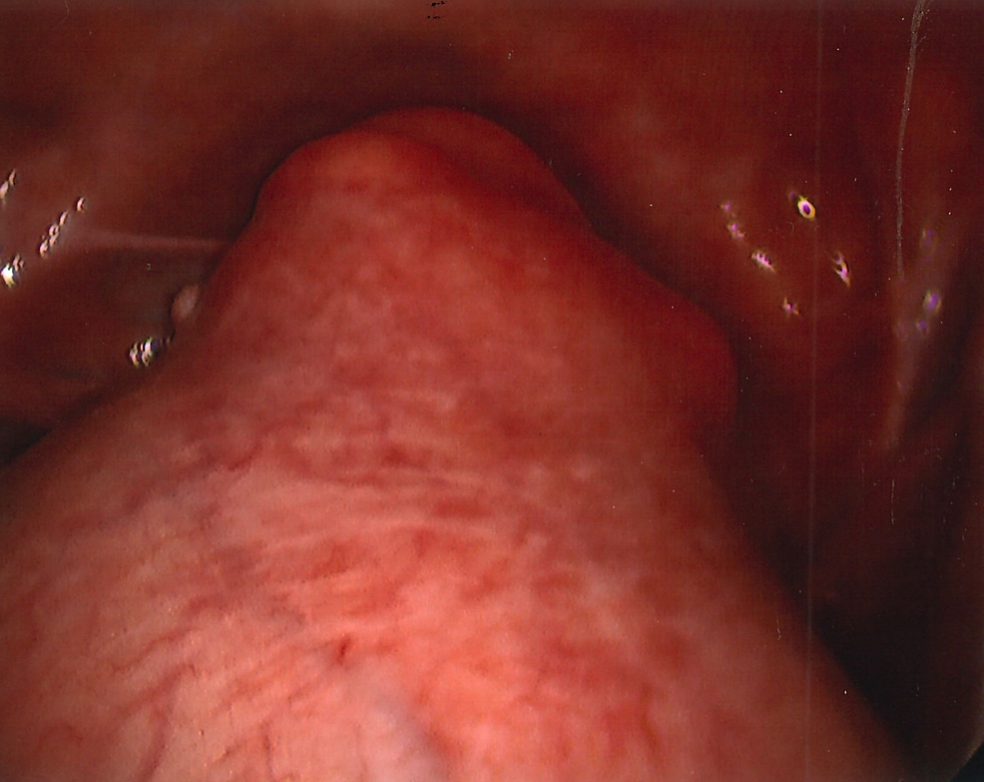
Laparoscopic view of fibroid uterus

**Figure 2:**
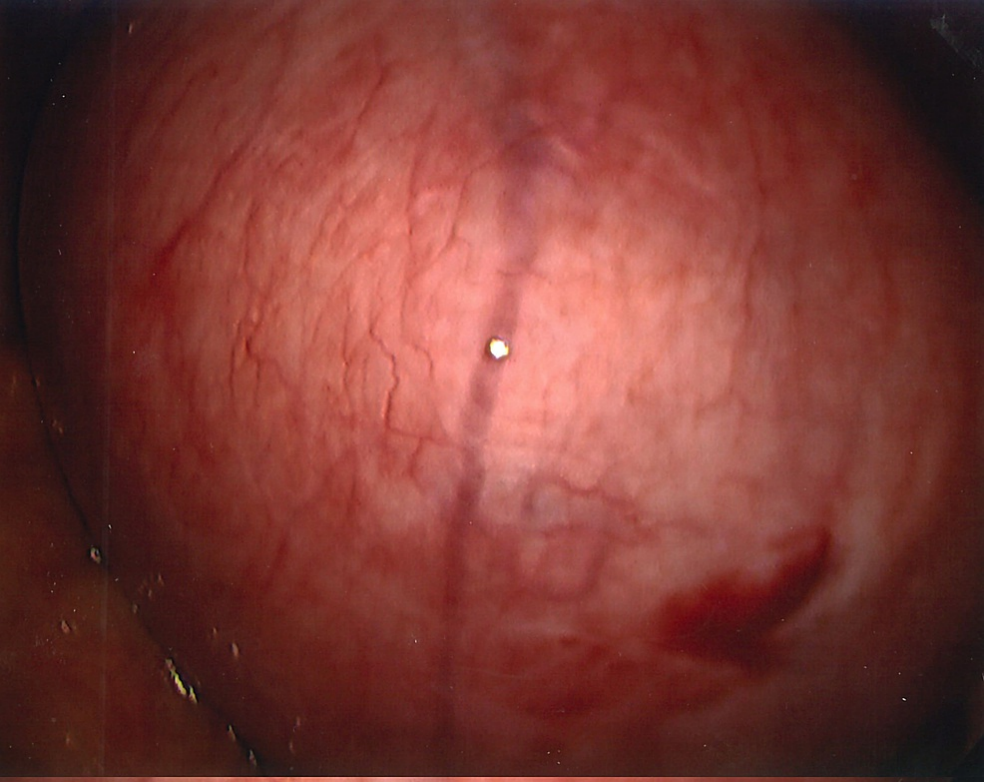
Laparoscopic view of fundal myoma

Ultrasound and subsequent saline-infused sonohysterographic mapping through this new office revealed my uterus to have at least six fibroids, enlarged to the dimensions of a 14-week pregnancy (Figure 1). The largest was a dominant, exophytic intramural fibroid arising from the fundus measuring 7.0 cm x 5.5 cm (Figure 2). The rest varied in size and were scattered in the intramural and submucosal layers. My right ovary was enlarged with either ethmoid or dermoid cysts suspected.

The unusual and acute development of depressive mood while on 5-mg Norethindrone became intolerable, so 2 mg of oral Estradiol was added to my medication regimen. It was the strangest shift in mood as if a gray film fell upon the world. Those symptoms resolved within days.

A month later and before potential surgery could be discussed, I was accepted into medical school in a new city, and my new life took over. The Estradiol/Norethindrone combination was off-label and not completely covered by my insurance. I paid out-of-pocket, but it improved the menorrhagia. I was petite and, to the casual observer, appeared lopsided back then. The left side of my pelvis bulged firmly almost to the level of my umbilicus, and it was like carrying a rock. Never one to miss an opportunity to learn and delight in knowledge, so my fellow students and I gently palpated the area. The pathologic and gross distortion was evident even to us. It became difficult to sit upright. There were night sweats, urinary frequency, pelvic pressure, proctalgia, and early satiety. Vigorous physical activity triggered the passage of clots regardless of where I was in my cycle.

I followed up with a new gynecologist to get back on track with symptom management and reassessment. A transvaginal and transabdominal ultrasound revealed no changes. We discussed options for intervention, of which it was clear that pharmacologic management would not be any more helpful, given their large size. When it came to discussing surgery, my primary goal was a short length of recovery. I asked whether a minimally invasive partial hysterectomy was an option since I never wished to have children. To my gentle frustration, the gynecologist was firm on not wishing to perform sterilization on a woman of childbearing age despite my personal preference. He did offer a minilaparotomy myomectomy, for which recovery would take four to six weeks: a near impossible length of downtime to find as a medical student. I had never been so busy in my entire life.

The opportunity came in the summer between my second and third year in May 2020. My school's regional campus model meant I needed to move to a new city just before the start of the third year curriculum. If a friend could help out in the weeks following, then a myomectomy would be doable. The first wave of the COVID-19 pandemic, however, caused delays. I canceled the surgery and moved before finding new housing would be difficult. Any surgical intervention would have to wait until the pandemic was over, and there was more leisure time as a fourth-year student. I used a telemedicine company to switch to 0.35-mg Norethindrone-only oral contraception that was fully covered under insurance and charged into my clinical year.

By this time, my periods fluctuated between spotting one month and pre-contraceptive levels of bleeding another month. Night sweats resolved for the most part. The sensation of pelvic heaviness, bloating, and pressure became increasingly noticeable. Urinary retention occurred with some cycles, but the urinary frequency was constant. It was not unusual to get up twice a night to empty my bladder. I timed my liquid consumption to ensure not to visit the restroom too often during clerkships. I tried not to drink before shelf exams, let alone while sitting for Step 1 and Step 2.

After completing my core clerkships and with a busy OB-GYN rotation under my belt, I possessed significantly more clinical and practical knowledge about my disease and treatment options. I rotated at clinics and with gynecologists who performed hysterectomies on reproductive-aged women if that was their well-informed desire. I assisted in more than one of those myself. I used my experience to listen. My brief mood interlude on high-dose progesterone allowed me to be more mindful when patients remarked upon their own sensitivities to types of birth control. By now, I was 37 years old and still G0P0. I still possessed no interest in having my own children. With this reinvigorating contextualization, I convened with local physician friends, faculty, and my primary care physician and was referred to the gynecologist of my choice.

Now, four years after my initial diagnosis, the process of establishing care to re-imaging to scheduling surgery was limited only by juggling residency interviews and a busy holiday season. Faculty accommodated by making small-course schedule adjustments to ease into a two-week-long winter break. This gave me a total month to undergo intervention and recover before the next rotation. That October, I was able to discuss my presentation, prior ultrasound, medication regimen, and treatment preference with ease together with my physician. Despite my second trimester-sized uterus, I was a good candidate for a laparoscopic-assisted partial hysterectomy. And with several negative cervical screenings, normal endometrial thickness, and stable fibroid size on imaging, contained power morcellation of the uterus was an option I was comfortable with. Shared decision-making happened at its finest.

My gynecologist recommended in the meantime to “Relax and get fit for surgery.” So I did.

**Figure 3:**
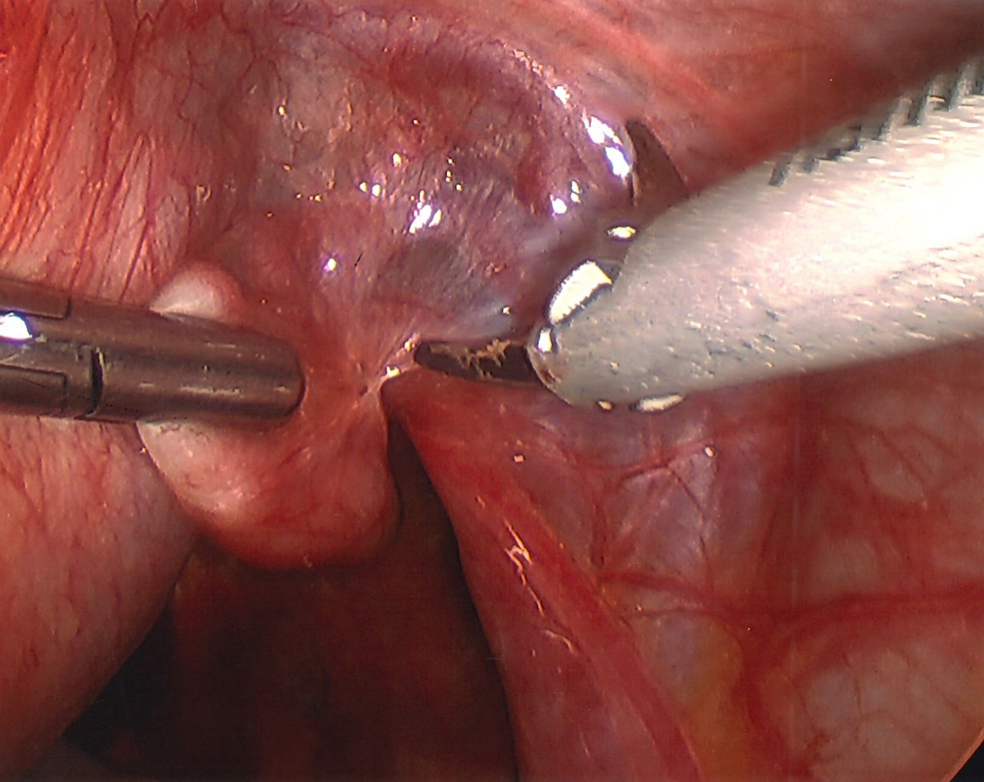
View of left ovary encased in scar tissue

**Figure 4:**
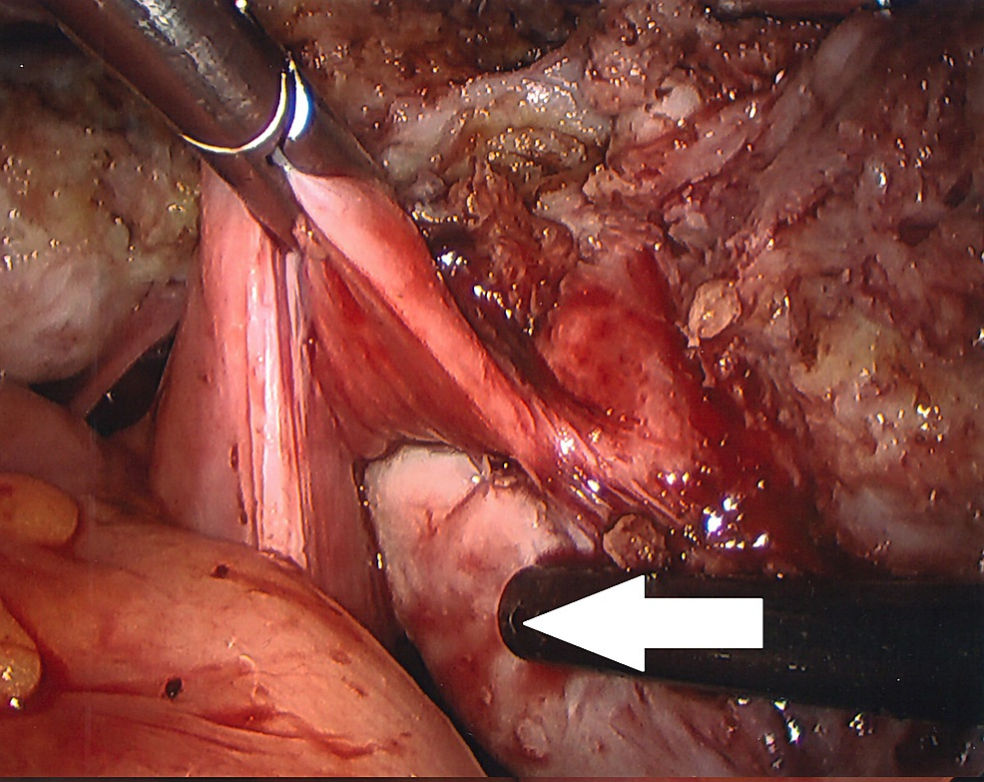
View of adhered right endometriotic ovary (arrow)

**Figure 5:**
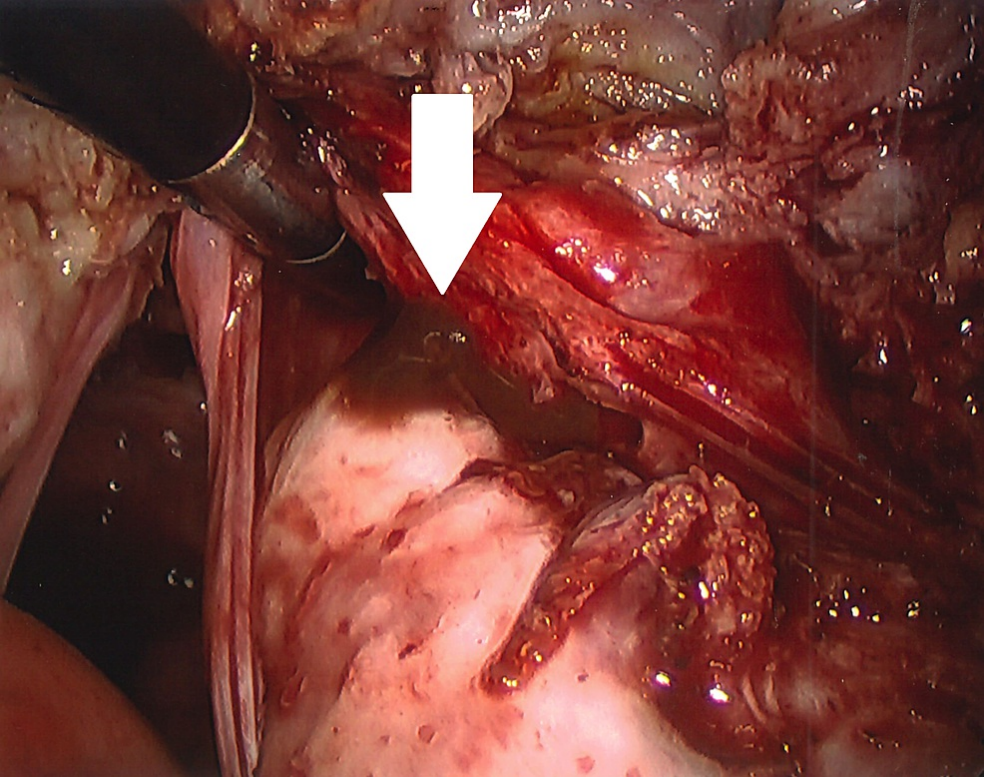
View of adhered right endometriotic ovary with characteristic chocolate fluid (arrow)

In December 2021, I underwent a laparoscopic-assisted supracervical hysterectomy with morcellation and bilateral salpingectomy. There was an impromptu addition of a right oophorectomy and lysis of adhesions of the bowel to the right ovary with the help of general surgery. The left ovary was very close to the cervix and encapsulated in adhesions (Figure 3). There was also severe endometriosis with severe adhesion toward the bowel and the pelvic region (Figures 4, 5). The general surgeon took that piece of bowel down, lysed the adhesions, and examined further to reveal the culprit as a suspected fold of peritoneum. For all its drama and suboptimal condition, the right ovary was sacrificed. The size of my uterus, the shift in structures, and the unforeseen endometriosis made visualization difficult but not impossible (Figures 1, 2). A testament to the skill of all involved, the procedure lasted approximately four hours and remained laparoscopic-assisted with only an additional 5-mm trocar hole to better see the colon. Total blood loss was 400 mL.

I awoke in the post-anesthesia care unit (PACU). Awareness rose in whispers and, in that silence, I brushed a hand over my abdomen. Empty. Empty, empty, empty. I was flat. It was gone. The absence of that firm, hard, boulder just to the left of and below my belly button was deafening. I was free.

**Figure 6:**
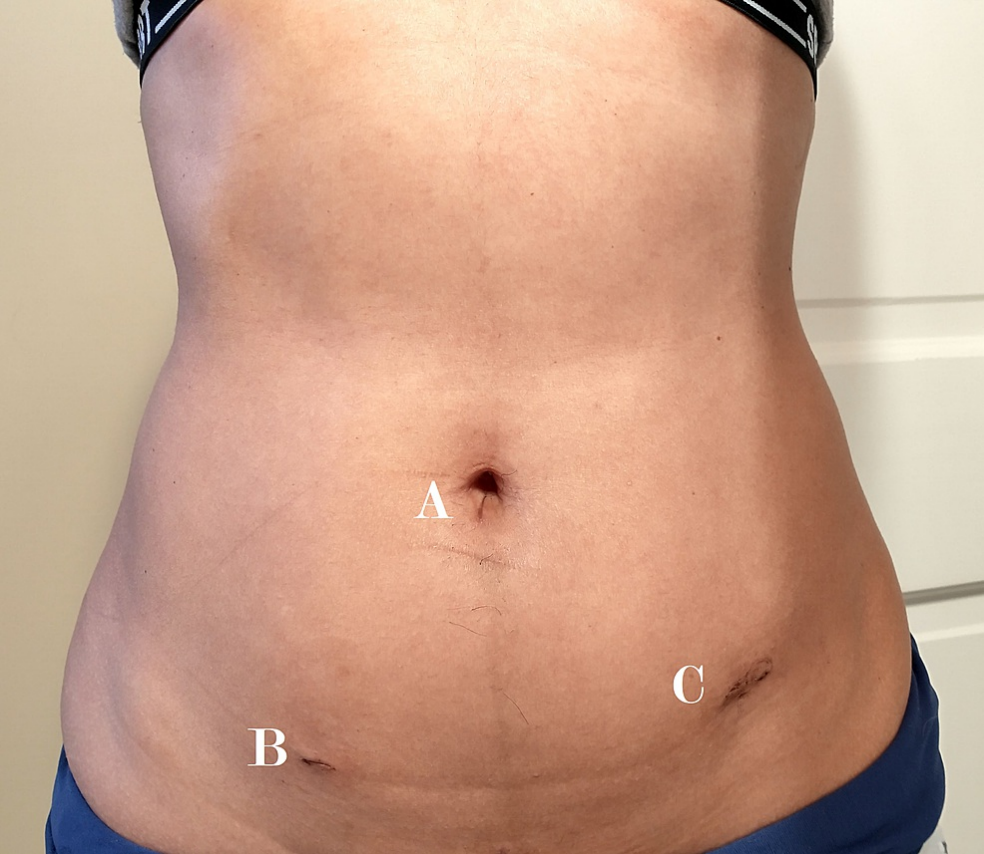
Excellent cosmesis of trocar incisions two weeks post-operatively (A) 5 mm subumbilical. (B) 5 mm right, inferolateral to the umbilicus. (C) 12 mm left, inferolateral to the umbilicus (5 mm suprapubic incision not pictured).

In the three months between my pre-op consult and my day-of-surgery check-in, I exercised approximately 290 minutes a week with combined stationary bike sessions and daily dog walking excursions. The relative youth and good physical conditioning afforded me a rapid post-operative recovery. The overnight stay was uneventful; I ate less than two hours later, walked shortly after that, and polished off Thomas Harris’ "The Silence of the Lambs" novel. Over-the-counter medication was all that was needed after post-op day 2 to address the odd discomfort of a new internal arrangement and the tiniest, cleanest trocar incisions I had ever seen (Figure 6, A-C).

Words fail to convey the unbearable lightness of no longer harboring a veritable rock in one’s pelvis. Within a week, I was walking a mile a day. In two weeks, I was walking three miles daily. By the third week, I was ready to get back to light biking and cleared for it. I can sit up straight. I can go the night without using the restroom. My pants do not pinch. I can run freely and fast. My body is my own.

Pharmacologic management

I was treated with oral Norethindrone 5 mg daily and oral Estradiol 2 mg daily for three years before being transitioned to oral Norethindrone 0.35 mg oral daily.

Surgical management

The procedure performed was a laparoscopic-assisted supracervical hysterectomy with bilateral salpingectomy and right oophorectomy and lysis of adhesions of the bowel to the right ovary.

**Figure 7:**
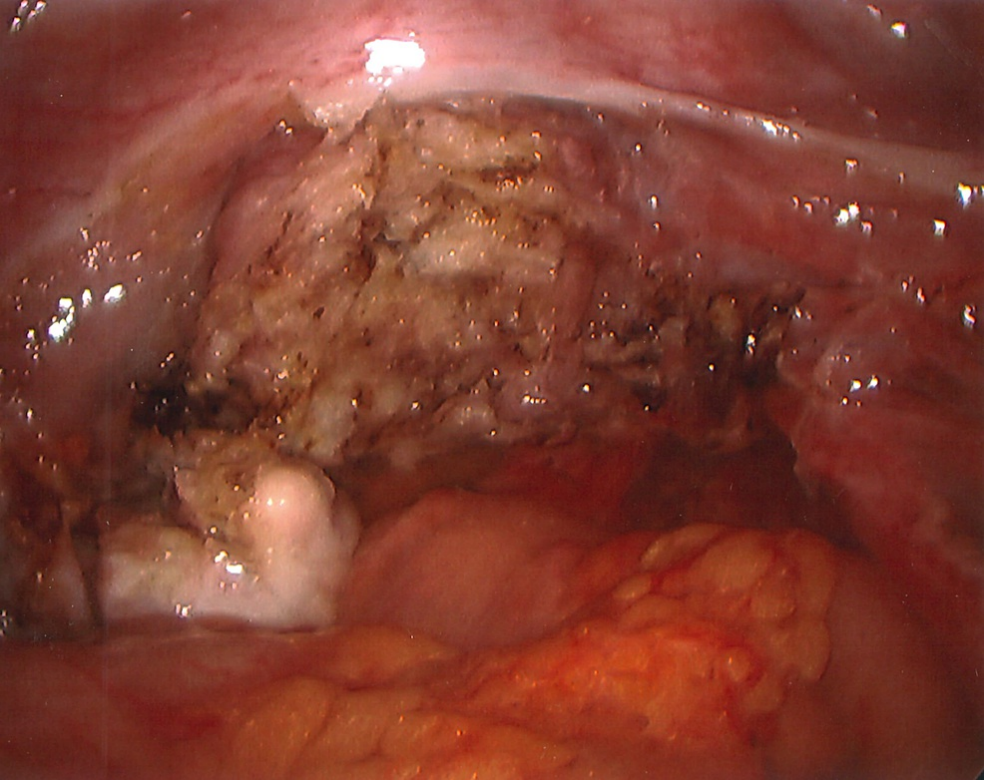
View of cleared operative field with completed LASH cervical stump LASH, Laparoscopic supracervical hysterectomy.

View of the posterior cul-de-sac was obfuscated by my large fibroid uterus (Figure 1). Some of the larger myomas (Figure 2) made initial visualization of the field difficult, so a 30-degree scope was used. A spleen bag was introduced into the abdominal cavity. The uterus, fallopian tubes, and right ovary were placed inside and morcellated, with the bag and its contents successfully removed. Figure 7 shows the cleared operative field and cervical stump. 

## Discussion

This case history describes the symptoms and barriers involved in diagnosing and treating uterine leiomyomas in an African American woman of childbearing age of low socioeconomic status. Without the incidental remark, while modeling for ultrasound, I might still be struggling with menometrorrhagia and numerous bulk symptoms. The robust health insurance and social support of medical school afforded me the relative luxury of undergoing a minimally invasive hysterectomy by the gynecologist of my preference. Experiences and a well-rounded education empowered me to self-advocate. Each piece laid the foundation needed to pursue treatment for this common and highly variable gynecological condition.

Despite their relatively benign pathology, uterine leiomyomas can present a significant emotional and economic burden to those afflicted. African American women, in particular, have more severe dysmenorrhea, mass symptoms, higher rates of hospitalization, myomectomies, and hysterectomies compared to their non-white cohorts [4-6].

Patients are individuals, so, with respect to the vast array of treatment options available, shared decision-making and informed consent are crucial. In this condition, the nature of the symptoms (or lack thereof) dictates treatment choice [5]. The disruption inherent to procedural interventions can pose a barrier to those without appropriate financial and emotional support. For the patient who does not wish to preserve fertility, physicians should maintain a patient-centered approach with consideration of hysterectomy versus myomectomy. The literature reflects numerous cases of large 12+ week-sized fibroid uteruses being amenable to laparoscopic-assisted myomectomy or hysterectomy, including contained morcellation [7-10]. Physicians and patients must be made aware of the relatively low risk (1%-2%) of sarcoma in leiomyomas [11], updated American College of Obstetricians and Gynecologists (ACOG) committee opinion summary [12], and the 2020 FDA recommendation for power morcellation only with a tissue containment system [13].

Scant published literature exists on the incidence of both uterine fibroids and endometriosis. However, my intraoperative findings show it to be possible. Leiomyoma patients may benefit from consideration of concomitant endometriosis. Dysfunctional uterine bleeding and pelvic pain can be of multifactorial origin, and both conditions share multiple features [14]. A 2021 Taiwanese study, using 14 years of data and over 150,000 women, revealed a statistically significant association in patients with uterine leiomyoma developing endometriosis (adjusted hazard ratio: 6.44) [15]. This and similar, smaller studies highlight an opportunity to increase awareness. Early diagnosis and treatment of endometriosis may improve the patient outcomes by mitigating some of the more distressing symptoms of pelvic discomfort, bloating, and dysmenorrhea before operative intervention is necessitated.

## Conclusions

Uterine leiomyomas are a very common gynecological condition. Challenges to its diagnosis and treatment continue to exist, and the literature reveals opportunities for patient ethnicity to be considered when assessing risk factors, presentation, and disease severity. For those with no interest in preserving fertility, childbearing age should not be a barrier in offering minimally invasive ovary-sparing hysterectomy. Laparoscopic supracervical hysterectomy remains a well-tolerated and low-risk option. The increased emotional, physical, and financial burden of treatment in addition to patient goals and surgeon expertise all play important roles in selecting the appropriate treatment and improving outcomes.
